# Role of Muscle Synergies in Real-Time Classification of Upper Limb Motions using Extreme Learning Machines

**DOI:** 10.1186/s12984-016-0183-0

**Published:** 2016-08-15

**Authors:** Chris Wilson Antuvan, Federica Bisio, Francesca Marini, Shih-Cheng Yen, Erik Cambria, Lorenzo Masia

**Affiliations:** 1 School of Mechanical and Aerospace Engineering, Nanyang Technological University, Singapore, Singapore; 2 Department of Naval, Electrical, Electronic and Telecommunications Engineering, University of Genoa, Genoa, Italy; 3 Department of Robotics, Brain and Cognitive Sciences, Italian Institute of Technology, Genoa, Italy; 4 Department of Electrical and Computer Engineering; Singapore Institute of Neurotechnology (SINAPSE), National University of Singapore, Singapore, Singapore; 5 School of Computer Science and Engineering, Nanyang Technological University, Singapore, Singapore

**Keywords:** Electromyography, Myoelectric control, Muscle synergy, Pattern recognition, Real-time control

## Abstract

**Background:**

Myoelectric signals offer significant insights in interpreting the motion intention and extent of effort involved in performing a movement, with application in prostheses, orthosis and exoskeletons. Feature extraction plays a vital role, and follows two approaches: *EMG* and *synergy* features. More recently, muscle synergy based features are being increasingly explored, since it simplifies dimensionality of control, and are considered to be more robust to signal variations. Another important aspect in a myoelectrically controlled devices is the learning capability and speed of performance for online decoding. Extreme learning machine (ELM) is a relatively new neural-network based learning algorithm: its performance hasn’t been explored in the context of online control, which is a more reliable measure compared to offline analysis. To this purpose we aim at focusing our investigation on a myoelectric-based interface which is able to identify and online classify, upper limb motions involving shoulder and elbow. The main objective is to compare the performance of the decoder trained using ELM, for two different features: *EMG* and *synergy* features.

**Methods:**

The experiments are broadly divided in two phases *training/calibration* and *testing* respectively. ELM is used to train the decoder using data acquired during the calibration phase. The performance of the decoder is then tested in online motion control by using a simulated graphical user interface replicating the human limb: subjects are requested to control a virtual arm by using their muscular activity. The decoder performance is quantified using ad-hoc metrics based on the following indicators: motion selection time, motion completion time, and classification accuracy.

**Results:**

Performance has been evaluated for both offline and online contexts. The offline classification results indicated better performance in the case of *EMG features*, whereas a better classification accuracy for *synergy feature* was observed for online decoding. Also the other indicators as motion selection time and motion completion time, showed better trend in the case of synergy than time-domain features.

**Conclusion:**

This work demonstrates better robustness of online decoding of upper-limb motions and motor intentions when using synergy feature. Furthermore, we have quantified the performance of the decoder trained using ELM for online control, providing a potential and viable option for real-time myoelectric control in assistive technology.

**Electronic supplementary material:**

The online version of this article (doi:10.1186/s12984-016-0183-0) contains supplementary material, which is available to authorized users.

## Background

Electromyogram (EMG) signals are nowadays the most widely used biometric information to translate human motion intention into action. Their main use ranges from interfaces in human-machine interaction based applications like prosthesis [1–3], orthosis [4–6] and tele-manipulation [7–10], to functional electrical stimulation as well [11, 12]. Myoelectric signals provide information like the intent and extent of motion, simplified synergistic model of motion control, and on biomechanics at the joint level as the impedance characteristics. As much as they offer useful information, they are also challenging to use in motion control, due to the fact that EMG signals are non-repetitive, and are subjected to degradation due to change in skin conductivity i.e. sweat, muscle fatigue and shift in electrode position. The two important factors detrimental to the performance of any decoding algorithm are: the feature extraction techniques applied, and the learning algorithm used to build the decoder relating the input (EMG signals) to the corresponding output (control-motions).

Feature extraction techniques are used to characterize the EMG signals and extract useful information, in order to be able to interpret them better. Two main approaches are the most widely used (Ison et al. [13]): a first one named *EMG features* relying on structural characteristics from each individual EMG channel (like time-domain, frequency-domain, time-frequency domain characteristics [14, 15]), and a second one namely known as *synergy features* which uses information from multiple EMG signals. Time-domain features are computationally simple to evaluate [16], and provide good levels of decoding accuracy, whereas frequency and time-frequency domain analyses foresee a higher computational process and furthermore they do not significantly increase the decoding accuracy when compared with the previously mentioned features [17]. *Synergy features* extract coordination patterns across multiple EMG channels, by means of time-variant or time-invariant synergies [18, 19]. Muscle-synergy is hypothesized to be the method by which brain simplifies motor control, and synergistic muscle activation patterns have been observed while performing specific movements, e.g. reaching tasks [20]. These features showed to be robust and not sensitive to amplitude cancellations, and also help control strategy simplification by reducing the control dimensionality [21]. Furthermore, synergy features have shown to be consistent and robust to slight shift in electrode position [22].

Apart from feature extraction techniques, the type and amount of training data used also does affect the performance of the decoder. Incorporating dynamically varying data [23] and including multiple limb positions [24] have shown to improve decoding performance, but causes an increase in the training data-set and ultimately leading to higher computational burden. Learning algorithms like artificial neural networks (ANN), linear discriminant analysis (LDA), support vector machines (SVM), and Gaussian mixture models have been extensively used in myoelectric-based motion control, and they provided good levels of accuracy. Nonetheless the learning efficiency and rate of classification should be fast enough for effective utilization in real-time applications. Extreme learning machine (ELM) is a relatively new supervised learning algorithm and represents a single-hidden layer feed-forward neural network (SLFNN) [25]. The learning rates are significantly higher than the traditional back-propagation based learning machines, and it provides an efficient solution to generalized feed-forward networks. ELM offers faster rates of training, less degree of intervention and ease of implementation. Shi et al. [26] shows that the running time of ELM is much faster than LDA and SVM; also, the results indicate the classification accuracy of ELM is overall higher than LDA, and almost comparable with that of SVM, thus showing the potential of ELM for real-time myoelectric control of assistive devices.

The main focus of this paper is to compare and contrast the performance of ELM using the two approaches *EMG features* (more specifically time-domain features) and *Synergy features*; this study will focus on the differences between the two approach in decoding shoulder and elbow motions. Decoding strategies could be either regression or classification: regression based decoding strategies are mostly employed in controlling devices, which are mainly aimed at augmenting the capability in either healthy or weak subjects, by amplifying human force/torque [27, 28]. In some cases, a neuromusculoskeletal model is used to relate EMG signals to torques produced at the joints, and the level of assistance to be provided by the device is set using a scaling factor [29, 30]. In the present paper we will focus on a classification model, since the target population is intended to be patients affected by brain injury with specific focus on stroke. In this kind of subjects, the use of regression models is particularly challenging due to the degraded EMG activity, with a resulting acquired signals often hard to interpret and uncontrolled muscular co-contractions (hypertonia) leading to unwanted torques generated by the device [27]. Hence we opted for an approach based on detection of a preset number of movements (classes) [31] where the decoder is devoted to classify the movements in real-time and control a graphical user interface replicating a human limb. Our hypothesis is that *synergy-features* could provide a more reliable and robust means of decoding myoelectric signals, and if proven initially in the case of healthy subjects, might translate reasonably to stroke population. Furthermore, synergies have been found to be either preserved, merged or fractioned in neurologically impaired subjects, depending on the extension of the brain lesion and the elapsed time from the stroke onset [32]. While ELM has been applied to determine performance measures in an offline context, we want to extend the experiment to online decoding, and determine how accurate ELM can fare in real-time control of assistive devices. This paper is a more extensive and comprehensive work than our previous contribution [33]. To our knowledge, no previous work have employed ELM to test the performance of the decoder for myoelectric control in an online scenario. In order to perform online decoding, a virtual avatar replicating a human limb has been implemented, and it enables a number of movement classes comprising shoulder and elbow degrees of freedom. The acquired EMG signals are processed in real-time, the classified outputs actuate the virtual avatar to provide feedback to the user, and enables modification of user learning to improve the performance, and understand the inverse model of the decoder to better control the graphical interface. The main target is to implement the algorithm and be able to interface a soft wearable exosuit for the upper-limb that is being currently developed in the lab.

## Methods

### Subjects

A total of 7 healthy subjects (6 males and 1 female, age 26.85±1.57 years) participated in the experiment, and they all provided written informed consents. The procedures were approved by the Institutional Review Board at Nanyang Technological University.

### Experimental setup

The setup includes Wireless Electromyografic system (Trigno wireless, Delsys Inc.), which is used to record the surface EMG signals. A real-time Data acquisition board (Quanser QPIDe) is used to acquire the EMG signals as analog inputs, and the acquisition frequency has been set to 1 kHz. A MATLAB/Simulink based custom program is then used to interface the myoelectric signals, processing them and running the classification algorithm. A high level routine converts the decoder output into control signals to a simulated arm in the virtual environment, thereby providing visual feedback to the subject during the *testing phase*.

### Experimental protocol

**Fig. 1:**
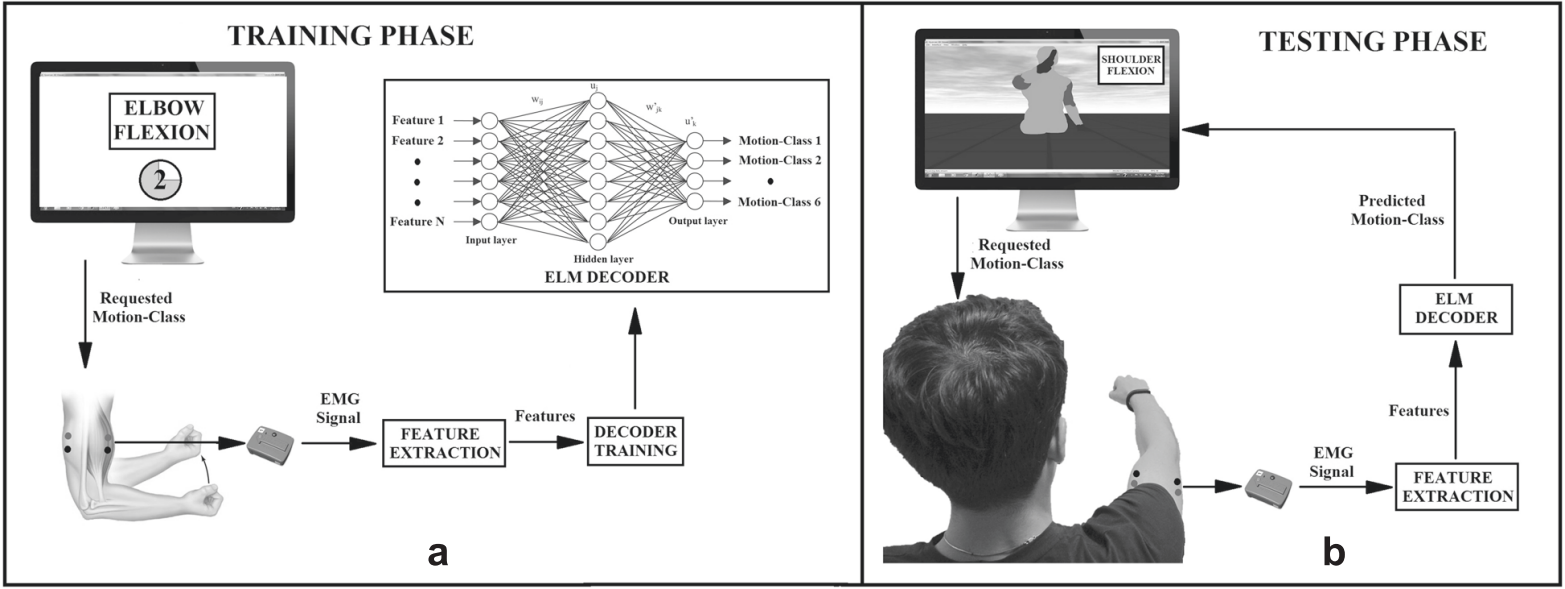
Training and Testing phase. **a** Represents the offline training phase, where the graphical user interface instructs the subject to perform specific movements for specific period of time. The EMG data is then used to build the decoding model which is specific for each subject. **b** The online testing phase, where EMG signals are decoded in real-time, and facilitates the movement of the virtual avatar

**Fig. 2:**
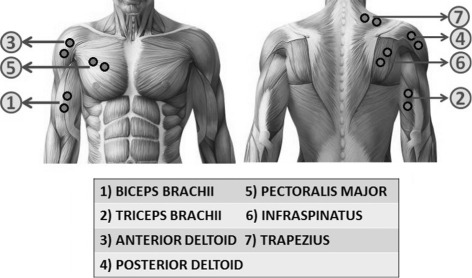
Muscles and electrode placement. Illustrates the muscles utilized in the experiment and the respective electrode placement on the muscles

**Fig. 3:**
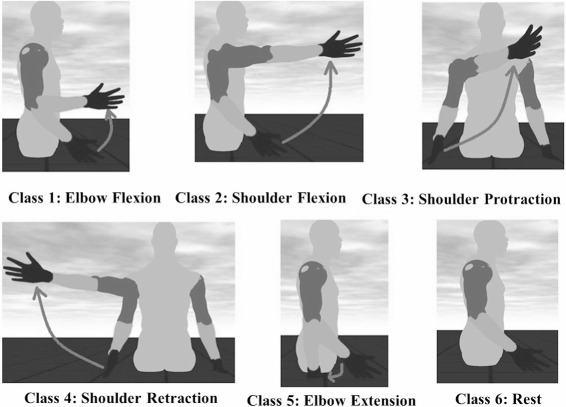
Motion-classes. The five different motion-class and the rest pose which represents the sixth class. The movement starts from the rest pose and transitions to the target pose as indicated in the figure, and motion is designed to follow a minimum-jerk trajectory

The investigation was divided into two experiments execute in two different days. Each experiment included two phases: a *training* and a *testing* phase respectively. Experiment 1 was performed using *EMG features*, while experiment 2 using *synergy feature*. Figure 1 shows the phases of the *training phase* as well as the online *testing phase*. EMG signals were recorded by seven channels and electrodes were placed on the muscles mainly responsible for execution of the shoulder and elbow movements involved, and are as shown in Fig. 2. The five different *motion-classes* involved in the experiment are as shown in Fig. 3.

During the *training phase*, subjects were instructed to perform all the *motion classes* and each of them is repeated ten times, for a total fifty repetitions; in this phase the main purpose is to acquire the EMG signal corresponding to the different movement classes. A graphical user interface indicates the movement (class) to be performed (trial) with two seconds of rest between two consecutive movement trials (each 3 s). A ten seconds resting phase is provided upon completion of ten repetitions for the same class. The EMG signals acquired during the *training phase* are used to train the decoder for the successive online *testing phase*.

During the *testing phase*, the subject’s EMG activity is detected and used to tele-operate a virtual avatar in the graphical user interface: the purpose is to allow the subject to learn the inverse model of the decoder and control the virtual model: in a scenario where an assistive device is used, the virtual avatar is intended to be a training interface before the user wears the device itself. The motion planning of the virtual arm is implemented using the minimum-jerk criteria [34]: after the graphical interface provide the instruction by indicating the name of the class, subjects were requested to move their arm in such a way the decoder classifies the movement and reproduce it on the simulated avatar. Each *motion-class* is performed twenty times, and the instructions are provided in a randomly generated sequence. Each trial is considered to be completed upon successful transition of the virtual arm, from the rest pose to the respective *motion-class* indicated, and holding the position for 0.5 s. There is no time restriction in completing the task, and a trial is considered successfully completed when the virtual movement matched the one requested.

### Feature extraction

All the EMG signals are pre-processed by rectification, followed by low-pass filtering with a cut-off frequency of 10 Hz, in order to obtain the envelope of the signal. Experiment 1 involves usage of *EMG features*, whereas experiment 2 uses *synergy features*, for online control of the graphical user interface. The input signals are represented by $ X ~\epsilon ~\mathbb {R}^{p \times q} $, where p and q are the number of original dimensions (number of EMG channels) and the number of data samples respectively. The features used are as mentioned below.

**EMG features:** We considered two time-domain features to extract information from the EMG signals [35], with a sliding window of 100 samples (100 ms) and an overlap of 90 samples between two windows to account for processing time: which means an output is produced every 10 ms.

*Mean Absolute Value (MAV)*: It is evaluated by taking the average of each EMG signal in the window frame, providing information on movement duration and effort [36].

1 $$ { MAV = \frac{1}{N}\sum_{n=1}^{N}\vert{x_{n}}\vert}   $$

where *N*=100 is the number of samples in the sliding window.

Variance (VAR): It is a measure of the EMG variability, and is an indicator of the EMG signal power and helps in identifying movement onset and contraction [36].

2 $$ { VAR = \frac{1}{N-1}\sum_{n=1}^{N}\vert{x_{n}}^{2}\vert}   $$

For each of the seven EMG channels, these two set of features are extracted, and hence the dimensionality of the input signals become fourteen. The input matrix after the *EMG features* extraction procedures, is represented by $ Y_{EMG} ~\epsilon ~\mathbb {R}^{2p \times q} $, where p and q are the number of original dimensions (EMG channels) and the number of data samples.

**Synergy feature:** Non-negative matrix factorization (NMF) is the decomposition technique used to transform the EMG signals from the muscle activity space to synergy space. It constrains the activation coefficient to be a non-negative value, which reflects the reality of the neural and muscle activation (pull-only behavior). Any non-negative matrix $ X ~\epsilon ~\mathbb {R}^{p \times q} $ is decomposed into two non-negative factors $ A~\epsilon ~\mathbb {R}^{p \times k} $ and $ Y~\epsilon ~\mathbb {R}^{k \times q} $, where p,q and k are the number of EMG channels, number of data samples, and the number of reduced dimensions respectively. The relation is given by

3 $$ { X = AY + E}  $$

$${} \begin{aligned} \left[\! \begin{array}{cccc} x_{11} &. & x_{1q}\\. &. &. & \\ x_{p1} &. & x_{pq} \end{array} \!\!\!\!\right] = \left[ \begin{array}{cccc} a_{11} &. & a_{1k}\\. &. &. \\ a_{p1} & & a_{pk} \end{array} \!\right] \left[ \begin{array}{cccc} y_{11} &. & y_{1q}\\. &. &. \\ y_{k1} & & y_{kq} \end{array} \!\right] + \left[ \begin{array}{cccc} e_{11} &. & e_{1q}\\. &. &. \\ e_{p1} & & e_{pq} \end{array} \right] \end{aligned} $$

where *E* is the error or residual. Each column in the matrix *X* represents multi-variate data points. *A* is the *basis matrix* containing the basis vectors of the synergy space, and *Y* is called the *coefficient matrix* containing the activation coefficients. The optimization technique used to converge to a stationary point is based on the *non-negative least squares* method proposed in [37]. The number of basis vectors is chosen based on the explained variance of the data or by the residual error *E* obtained by approximation. In this experiment, the number of basis/synergy vectors to be used is based on two criteria: firstly, to ensure that at least 90 *%* of the variance in the data could be represented using the reduced dimension and secondly, the point in the cumulative explained variance plot where the change in slope is less than 5 *%* of the variance.

During the *training phase*, the input matrix (*synergy feature*) obtained after performing the decomposition algorithm is represented by the *coefficient matrix*$ Y_{SYN} ~\epsilon ~\mathbb {R}^{k \times q} $, where k and q are as mentioned above. During the *testing phase*, the *coefficient matrix* are obtained in real time by using the following relation.

4 $$ {Y_{SYN} = A^{-1} \times X}   $$

$$\begin{aligned} \left[ \begin{array}{cccc} y^{1}_{11} &. & y^{1}_{1q}\\. &. &. & \\ y^{1}_{k1} &. & y^{1}_{kq} \end{array} \right] = \left[ \begin{array}{cccc} a^{-1}_{11} &. & a^{-1}_{1p}\\. &. &. \\ a^{-1}_{k1} & & a^{-1}_{kp} \end{array} \right] \left[ \begin{array}{cccc} x_{11} &. & x_{1q}\\. &. &. & \\ x_{p1} &. & x_{pq} \end{array} \right] \end{aligned} $$

where $ X ~\epsilon ~\mathbb {R}^{p \times q} $ represents the multi-variate data points, $ A~\epsilon ~\mathbb {R}^{p \times k} $ represents the *basis matrix* obtained during the *training phase*, and $ Y_{SYN}~\epsilon ~\mathbb {R}^{k \times q} $ represents the input matrix after performing synergy-space feature extraction.

### Machine learning algorithm

We decided to use Extreme Learning Machine (ELM) for decoding the motion classes and consequently controlling the virtual arm model. ELM is an emerging learning paradigm that represents an efficient unified solution to generalized feed-forward neural networks including (but not limited to) single-/multi-hidden-layer neural networks, radial basis function networks, and kernel learning. ELM offers significant advantages such as fast learning speed, ease of implementation, and minimal human intervention [38]. It thus has strong potential as a viable alternative technique for large-scale computing in many different applications, including image [39], text [40], speech [41] and multimodal [42] processing, but also cognitive learning [43] and reasoning [44].

The ELM model [45] implements a single-hidden layer feedforward neural network (SLFNN) with *N* mapping neurons. The function connecting the input layer with the hidden layer can be expressed as follows, for each neuron *j*∈{1,…,*N*}:

5 $$ h_{j}(\mathbf{Y}) = a(\mathbf{Y},{R}_{j})   $$

where $\mathbf {Y} \in \mathbb {R}^{Z}$ is an input stimulus which is either *Y*_*EMG*_ or *Y*_*syn*_ depending on the type of feature used for the experiment, *a*(**Y**,*R*) is a nonlinear piece-wise continuous function (activation function), characterized by *R* which denotes the set of parameters of the mapping function. Every *j*-th neuron has its set of parameters *R*_*j*_.

The overall output function connecting the hidden and the output layer is expressed as

6 $$ f(\mathbf{Y}) = \sum_{j=1}^{N} \mathbf{w}_{j} h_{j} (\mathbf{Y})   $$

where **w**_*j*_ denotes the weight that connects the *j*-th neuron with the output.

The peculiar aspect of ELM is that the parameters *R*_*j*_ are set randomly. Hence, if one uses, for example, Radial Basis Functions to implement *a*(.):

7 $$ a(\mathbf{Y},{R}) = exp\left(-\zeta{\Vert{\mathbf{Y - c}}\Vert}^{2}\right)   $$

the parameters to be set randomly are the coordinates of each centroid, $\mathbf {c} \in \mathbb {R}^{Z}$, and the quantity *ζ*. In this study triangular basis function was used as the activation function.

Accordingly, the hidden layer implements an explicit mapping of the original input space into a new space $ \mathbb {R}^{N}$. Hence, training ELMs is equivalent to solving a regularized least squares problem in a linear space. Let $H \in \mathbb {R}^{n \times N}$ be an activation matrix such that the entry *H*_*i*,*j*_ is the activation value of the *j*-th hidden neuron for the *i*-th input pattern. Then, the training problem reduces to the minimization of the convex cost:

8 $$ \mathbf{w}^{*} = \arg \min_{\mathbf{w}} \left\| H \mathbf{w} - \mathbf{o} \right\|^{2} + \lambda \left\| \mathbf{w} \right\|^{2}   $$

where *λ* controls the contribution of the regularization factor.

The vector of weights **w**^∗^ is then obtained as follows:

9 $$ \mathbf{w}^{*} = (H^{T}H+\lambda I)^{-1}H^{T}\mathbf{o}   $$

where $I \in \mathbb {R}^{N \times N}$ is an identity matrix.

The ELM model can be conveniently described as a 2-stage learning machine. In the first stage, the data originally lying in the Z-dimensional space are remapped into a new N-dimensional space (ELM feature space) by exploiting as many ‘random’ neurons. Then, an RLS problem is solved for learning the linear classifier in the N-dimensional space.

The final decision function of ELM for a binary classification problem is:

10 $$ f_{L}(\mathbf{Y}) = sign (f(\mathbf{Y}))  $$

For multiclass problems, it is possible to set multi-output nodes: therefore, *m*-class classifiers have *m* output nodes. If the original class label is *c*, the expected output vector of the *m* output nodes for the *i*-th input pattern is *o*_*i*_=[0,…,0,1,0,…,0]^*T*^, with the *c*^*th*^ element of *o*_*i*_=[*o*_*i*1_,…,*o*_*im*_]^*T*^ set to one, while the rest of the elements are set to zero.

The classification problem for ELM with multi-output nodes can be formulated as:

11 $$ \underset{w}{min}\left\{{\frac{1}{2}}{\Vert\mathbf{w}\Vert}^{2} + C\frac{1}{2}\sum_{i=1}^{n}{\Vert\epsilon_{i}\Vert}^{2}\right\}  $$

Subject to: $h(\mathbf {Y}_{i})\mathbf {w} = {{o_{i}^{T}}}-{{\epsilon _{i}^{T}}} ~i=1,\ldots,n$

Where *ε*_*i*_=[*ε*_*i*1_,…,*ε*_*im*_]^*T*^ is the training error vector of the *m* output nodes with respect to the training sample **Y**_*i*_.

Therefore, the binary classification problem can be considered as a specific case of multi-output nodes when *m* is set equal to one. For both cases, the hidden layer matrix *H* remains the same, and the size of *H* is only decided by the number of training samples *n* and the number of hidden nodes *N*, which is irrelevant to the number of output nodes (number of classes) *m*.

### Performance metrics

In order to compare the accuracy of the ELM algorithm for *EMG features* and *synergy feature*, we evaluate the *offline performance* (corresponding to the *training phase*) and *online performance* (corresponding to the *testing phase*).

*Offline Performance* is defined as the accuracy of the decoder to classify the EMG patterns and associate them to the correct *motion-class*. The quantity of data samples collected during the training phase is split in two parts: training data-set which is used to train the decoder and its neural network, and the testing data-set which is used to test the performance of the decoder in predicting the *motion-classes*. A 2-fold cross validation procedure is used to determine the number of hidden layer neurons, and avoid over-fitting of the data. The testing data-set is used to determine the classification accuracy once the model parameters are optimized according to the following equations:

12 $$ {Acc}_{off} = \frac{n_{c}}{n_{c} + n_{w}} \times 100(\%)   $$

where *n*_*c*_ and *n*_*w*_ represents the number of correctly and wrongly classified samples respectively, from the testing data-set.

*Online performance*: It has the purpose of quantifying the accuracy of the algorithm during the online testing phase, where the subject is controlling the virtual arm by EMG signals and three of the indicators are based on the work by Li et al. [46] which are described below.

1. **Motion Selection Time**: It is the amount of time needed by the decoder to decode the human EMG patterns, translate them into motor commands to the virtual arm. It is mainly the time needed by the decoder to identify the desired *motion-class*.
2. **Motion Completion Time**: This is the amount of time subjects take to utilize their EMG signals and move the virtual arm into the desired position, and holding it for 0.5 seconds.
3. **Learning Trend**: It provides the trend of *motion completion time* through the experiment for each *motion-class*. The decreasing slope of the exponential fitting indicates a positive learning rate, provided by the general formula:
  13 $$ { y(x) = a\times e^{-bx} + c}   $$
  where *xε* {1,2,…,20} is the number of repetitions, *y* is the time taken to complete the task (a particular *motion class*) corresponding to *x*. While *a*,*b*,*c* respectively represent the initial performance, the learning rate, and the steady state value.
4. **Online Classification Accuracy**: It is used to calculate the classification accuracy of the decoder for each class. It is the ratio between the number of samples from the decoder matching the desired motion class and the total number of samples for that particular trial. Classification accuracy is calculated as:
  14 $$ {Acc}_{on} = \frac{1}{N}\sum_{i=1}^{N}\frac{n_{i}}{n_{i} + m_{i}} \times 100(\%)   $$
  where *N* represents the number of successfully completed trials in a particular *motion-class* for a subject. *n*_*i*_ and *m*_*i*_ represents the number of correctly and incorrectly classified samples respectively in a single trial *i*.
5. **Variability in muscle activity trends**: This is evaluated for each subject and for each *motion-class*, across all the seven EMG channels for both *EMG feature* and *synergy features*. It is the mean of the standard deviation values of the EMG activity across all EMG channels during the 20 repetitions of each *motion-class*. Each trial or repetition is normalized with respect to the completion time to indicate percentage of motion and resampled to a common set of 200 samples. The mean muscle activity and the deviation from the mean are calculated at each instant during the entire motion. It provides insight into the strategy used by the subjects and the performance consistency (precision) across the whole experiment and hence, the decoder performance.
  15 $$ Variability = \frac{1}{nEMG}~\frac{1}{nSamples}\sum_{i=1}^{nEMG}~\sum_{j=1}^{nSamples} \sigma_{ij}   $$
  where *nEMG*=7 represents the number of EMG channels, *nSamples*=200 represents the number of samples during each motion after resampling, and *σ*_*ij*_ is the standard deviation of the muscle activity for the *i*^*th*^ EMG channel and at the *j*^*th*^ time sample. The lower the variability value the better the decoder performance.
  16 $$ \sigma_{ij} = \sqrt{\frac{1}{nRep}\sum_{k=1}^{nRep}(E_{ij}^{k}-\mu_{ij})^{2}}   $$
  where *nRep*=20 represents the total number of repetitions for each *motion-class*, $ E_{ij}^{k} $ is the EMG activity for the *i*^*th*^ EMG channel at the *j*^*th*^ time sample and *k*^*th*^ repetition, and *μ*_*ij*_ is the mean of the EMG activity for the *i*^*th*^ EMG channel at the *j*^*th*^ time sample.

**Fig. 4:**
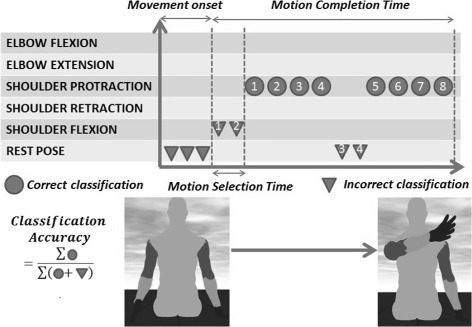
Performance metrics. Evaluation of the different metrics for a typical trial. An example of shoulder protraction trial is indicated in this figure in order to explain each metric. Subjects, starting from the rest position, are instructed by a message on the screen to execute a shoulder protraction. Movement onset initiates when decoder starts predicting the *motion-classes*. *Motion selection time* is defined as the interval between movement onset and decoder output which corresponds to the desired *motion-class*. *Motion completion time* is the time duration between movement onset and the final configuration of the virtual arm corresponding to the requested movement. *Classification accuracy* is the decoding accuracy throughout the trial

Figure 4 represents the various quantifying measures used for online decoding. A two-way Anova was performed to compare the statistical significance between *EMG features* and *synergy feature* for the five *motion-classes*.

## Results

### Decoder offline performance

Offline Decoder Classification accuracy has been evaluated for each subject across all the five different motion classes. The average accuracy in experiment 1 (*EMG features*) resulted in 99.37±0.81 *%*, whereas the average accuracy in experiment 2 (*synergy feature*) is 65.73±2.60 *%*. Based on a validation data-set, it resulted that the optimal number of hidden neurons for experiment 1 (*EMG features*) was 5000 units; contrarily 3500 units were chosen for experiment 2 (*synergy feature*), to avoid overfitting. The difference in the number of hidden neurons did not affect the processing time significantly during the testing phase. It took an average of 2.87±0.06 seconds to predict the output of 10000 samples in the case of *EMG features*, whereas it took around 2.01±0.02 seconds to predict in the case of *synergy feature*. Therefore, for real-time predictions, the difference in the number of hidden neurons would hardly impact the *motion selection time* or the *motion completion time*, especially because ELM is considered to have a very fast running time. The number of synergy vectors to be used in the experiment is based on two criteria, as mentioned previously. From the initial analysis it was determined that, using the first four synergy vectors matched the requirements, and was fixed for all the subjects to have uniformity. The average residual error obtained for all the subjects had an *R*^2^ value of 5.38±3.6 *%*.

### Decoder online performance

**Fig. 5:**
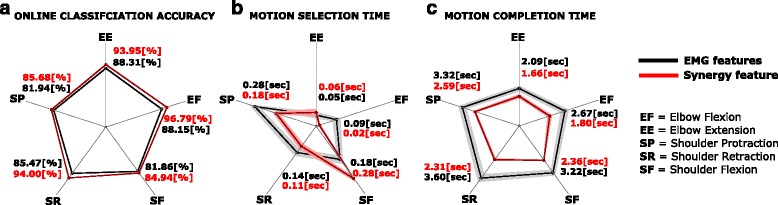
Decoder performance for each metric. Figures represent a polar plot indicating the mean and standard deviation for all the *motion-classes*, in the case of *EMG* and *synergy* features across all subjects. **a** indicates the plot of online classification accuracy, **b** indicates the plot of motion selection time, and **c** indicates the plot of motion completion time

**Fig. 6:**
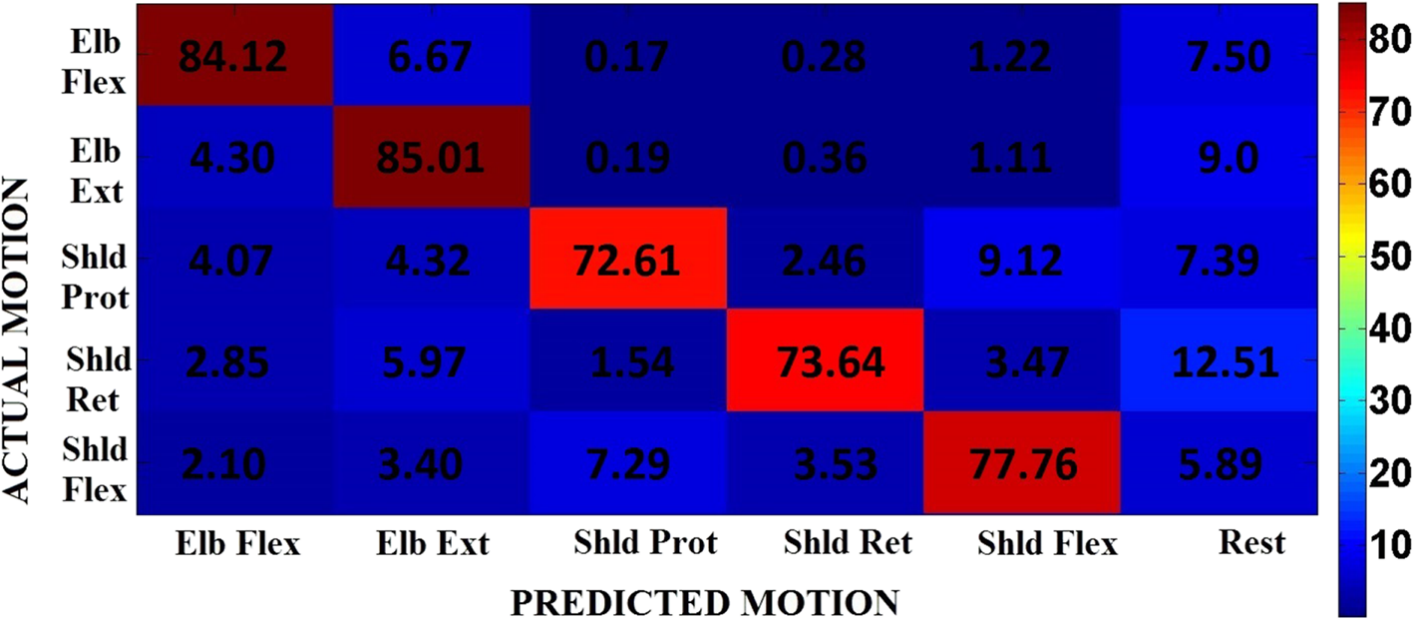
Normalized confusion matrix. Normalized confusion matrix across all subjects in experiment 1 when the decoder uses *EMG features*. This plot gives an idea of the misclassifications happening during decoding. The rows indicate the actual or requested *motion-class*, and the columns indicate the decoder prediction accuracy under each of the *motion-classes*, averaged across all the trials and subjects

**Fig. 7:**
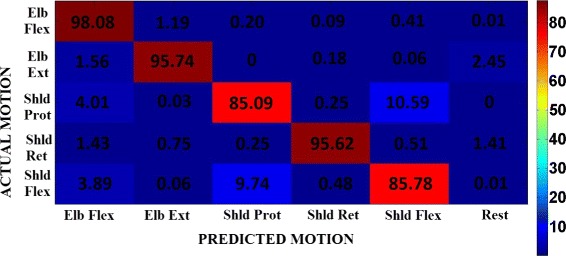
Normalized confusion matrix. Normalized confusion matrix across all subjects in experiment 2 when the decoder uses *synergy feature*. This plot gives an idea of the misclassifications happening during decoding. The rows indicate the actual or requested *motion-class*, and the columns indicate the decoder prediction accuracy under each of the *motion-classes*, averaged across all the trials and subjects

**Online Classification Accuracy:** The average classification performance of the decoder in the case of experiment 1 is 84.09±14.35 *%*, whereas in the case of experiment 2 is 91.79±9.86 *%*. The online classification accuracy reached with the *synergy feature* differs from the one reached with the *EMG features* with high statistical significance (two-way Anova, *F*_1,1284_=107.81,*p*<0.001), for all the five *motion-classes*. The classification accuracy for each *motion-class* and for both types of features are as shown in Fig. 5a. The confusion matrix of the classification accuracy is represented in Figs. 6 and 7 corresponding to *EMG features* and *synergy feature* respectively, and help to get a better understanding of the misclassifications in decoding.

Considering the metrics used to characterize online decoder performance during the *testing phase*, it was found that Motion Selection Time for experiment 1 was 0.15±0.21 seconds, while it was 0.11 ± 0.19 for experiment 2. A high significant difference was found between the two types of feature (*F*_1,1284_=12.428,*p*=0.00044); Anova test revealed a significant interaction between the type of the feature and the *motion-class* (*F*_4,1284_=14.265,*p*<0.001), in particular for elbow extension and shoulder retraction, the difference between *EMG features* and *synergy feature* did not reach significance (according to a post-hoc Fisher test). The selection time for each *motion-class* and for both types of features are as shown in Fig. 5b.

For Motion Completion Time the average time taken to complete each task across all the subjects and *motion-class*, in experiment 1 is 3.09±1.98 s, whereas it is 2.09±0.30 s in experiment 2. According to the two-way Anova test, there is a high significant difference (*F*_1,1284_=101.85,*p* < 0.001) between the classification accuracy between the *EMG features* and the *synergy feature*, and the difference is significant for all the five *motion-classes* as observable for Fig. 5c.

The Learning Trend values have been evaluated for each *motion-class* across all subjects and for both the experiments are as shown in Tables 1 and 2. A negative value indicates the occurrence of learning, leading to an improvement in performance, and contrary for the case when the value is positive. There is no consistent indication for learning trend across subjects and task, meaning that the decoder performance is strictly dependent on the features (EMG vs synergy) used for training and classification.

**Table 1:** Learning trend. Learning rate values for all the subjects for each of the *motion-class* in experiment 1 (*EMG features*)

Subject	Elbow flexion	Elbow extension	Shoulder protraction	Shoulder retraction	Shoulder flexion
Subject 1	−0.0019	0.0023	0.0029	0.0308	0.0150
Subject 2	−0.0074	0.0104	−0.0103	0.0233	−0.0150
Subject 3	−0.0381	−0.0187	−0.0373	−0.0499	−0.0194
Subject 4	0.0061	−0.0090	0.0347	0.0188	0.0101
Subject 5	0.0031	−0.0082	−0.0004	−0.0012	0.0138
Subject 6	0.0132	0.0096	−0.0010	−0.0047	0.0018
Subject 7	−0.0033	0.0034	−0.0045	−0.0170	−0.0075

**Table 2:** Learning trend. Learning rate values for all the subjects for each of the *motion-class* in experiment 2 (*synergy feature*)

Subject	Elbow flexion	Elbow extension	Shoulder protraction	Shoulder retraction	Shoulder flexion
Subject 1	−0.0000	0.0036	−0.0066	0.0002	−0.0004
Subject 2	−0.0000	−0.0120	0.0002	−0.0006	0.0029
Subject 3	−0.0156	0.0023	−0.0027	−0.0010	0.0032
Subject 4	−0.0009	0.0046	0.0029	−0.0001	−0.0040
Subject 5	−0.0007	0.0098	−0.0007	−0.0009	−0.0029
Subject 6	−0.0075	−0.0292	0.0002	−0.0001	−0.0008
Subject 7	−0.0046	0.0017	−0.0006	−0.0007	0.0018

**Fig. 8:**
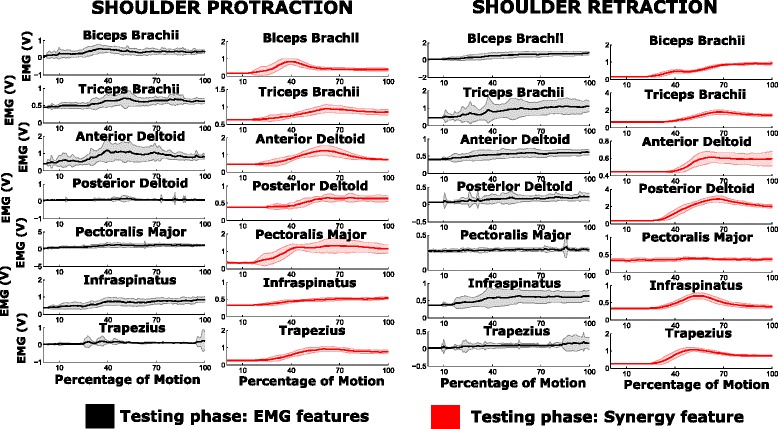
EMG activity during training and testing phase. Represents the mean and standard deviation plot of the EMG activity for subject 4 in each of the *motion-classes*. The plots in black and red pertains to the EMG activity during testing phase (20 repetitions) corresponding to *EMG features* and *synergy feature* respectively

**Fig. 9:**
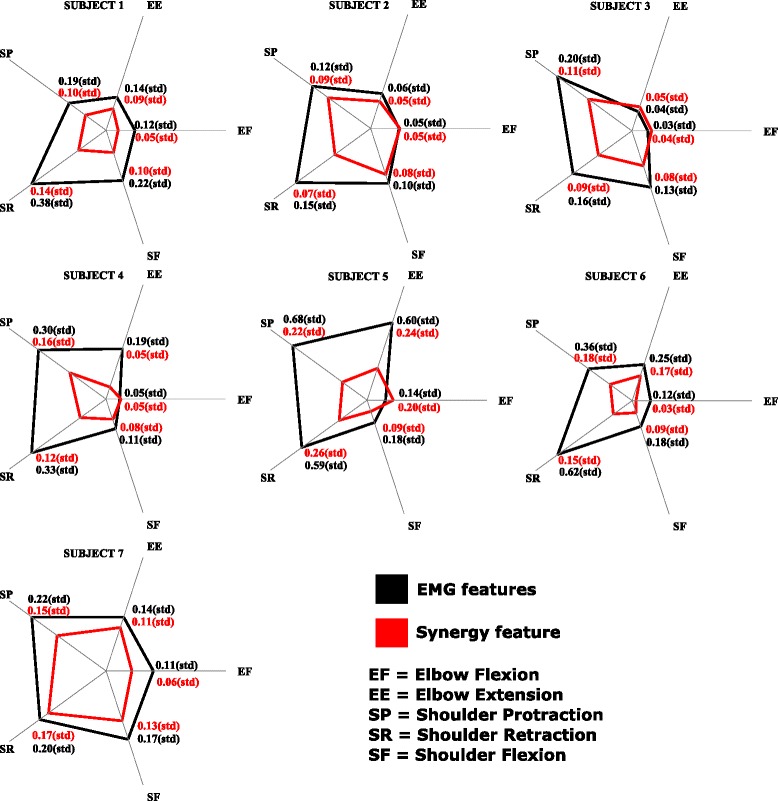
The average standard deviation of muscle activity. The mean of the standard deviation values of the muscle activity across all muscles during the entire motion (standard deviation values are as shown in Fig. 8) is indicated for each subject and each *motion-class*, in the case of *EMG features* and *synergy feature*

**Variability in muscle activity trends:** A typical muscular activation pattern in a subject when performing the shoulder protraction and shoulder retraction movements (multi-articular movements) is shown in Fig. 8. A reduced set of *motion-classes* are shown for better visual clarity and understanding. It can be observed that the EMG activity is smoother and less variable in the case of *synergy feature* decoded action, than in the case of *EMG features*. The variability values for each subject and for each *motion-class* are as shown in Fig. 9. The values are very high for the shoulder movements in the case of *EMG features* when compared to *synergy feature*, and the trend is pretty much similar for most cases in the elbow movements as well. A possible reason for the aforementioned difference might be in the presence of visual feedback provided to the subject by the virtual avatar, which was present in the testing phase and not in the training one. In the training phase the subjects were instructed to execute a movement and EMG channels were acquired to train the decoders. The testing phase consisted in asking the subject to perform movements corresponding to the selected classes and the results of the decoded movements were promptly shown as the decoded motion of the virtual arm in the visual feedback. There is a significant difference between the *EMG features* and *synergy feature* decoders in the case of online classification accuracy, with the *synergy feature* decoder performing better and allowing subject to control the virtual arm in a faster way (see motion completion time metric). The difference in online performance between the two decoders may alter considerably the muscular activation pattern by the subjects, who perceive the delay in decoding the desired movements provided by visual feedback. When instructed to move in a particular direction (*motion-class*) the participants can instantaneously perceive the efficacy of the decoder, and in the case of *EMG features* decoding, they tended to adjust by modifying the contraction patterns, and that’s why the EMG signals appear to be less smooth and present higher variability than in the case when *synergy feature* decoding was used.

## Discussion

In this paper our aim was to point out the differences in classification strategy using two approaches for feature extraction procedures. The offline classification accuracy indicated better performance in the case of *EMG features* when compared to the *synergy feature*, whereas one can observe an exact opposite trend in the case of online classification accuracy. This result is in line with other previous contributions showing that there is no correlation between offline and online classification accuracy [47, 48]. The main difference between the offline and online motion decoding is that in the second case, a real-time visual feedback of the user’s performance is provided. The visual feedback enables the subject to modify the neural activations, so as to recalibrate and account for the error in motion decoding. Real-time performance metrics are important in order to examine the robustness and accuracy of pattern recognition and eventually implement a control strategy able to precisely detect subject motor intention by means of EMG signals [46]. Therefore, the online classification metrics allowed to estimate the reliability of the decoder for both EMG and Synergy features and compare them. In the work by Jiang et al. [49], the authors claim that a perfect model relating muscle activity to control outputs is not essential, but rather continuous interaction and adaptation of the user with the myoelectric interface through feedback can help in achieving reliable performance. Irrespective of whether the model is intuitive or not, users are still capable of learning the inverse dynamics of the model itself, and its mapping function [50]. The level of intuitiveness of the model and the time taken for familiarization with the decoder, are factors which influence the improvement in performance. In our experiments, each motion-class was performed 20 times by each subject during the online testing, and yet we did not observe a consistent learning trend (Tables 1 and 2); which means that the optimum level of learning has not been reached yet: relatively to our primary target, which was the comparison between two extraction features, the absence of a learning trend or adaptation is not detrimental and does not affect the final results. Since the number of repetitions is constant in both the feature types, the absence of subjects adaptation implies that *synergy feature* based decoder is more effective than *EMG features*, and does not require familiarizing time in order to improve the performance; contrarily there might be the possibility that for higher number of trials the two decoders might converge to the same accuracy level, but still the *synergy feature* based decoder remains the optimal one, especially because it resulted to be more versatile and efficient in translating user biometric signals into motor command to the simulated virtual environment as reported in the previous sections.

Despite significant better online performance in *synergy feature* than *EMG feature*, there are still some cases in which the two decoders are comparable. Taking a closer look at the classification accuracy in the confusion matrix (Figs. 6 and 7) between the target class (requested to the subject) and the predicted class (decoded from subject movement) we can observe that the highest percentage of wrong classifications is in the decoding of shoulder protraction movement with a misclassification towards shoulder flexion movements and vice versa. A possible explanation could be found in the similarity between the two movements for what concern the muscular activation sequences during the executions forcing the decoder in misleading the prediction, which gets refined only when the execution has past the movement onset stage. Moreover, we can also notice that *motion-classes* involving shoulder movements have lower classification accuracy than the elbow movements in the case of *EMG features*: this effect is mainly due to the recruitment of multi-articular joints which involves a higher number of muscles compared to single joint (i.e. elbow), and hence the level of uncertainty in decoding increases.

However, in the present paper we have not addressed the challenge of simultaneous decoding of multiple *motion-classes* which is recently being explored by Young et al. and Jian et al. [49, 51] on groups of healthy subjects and trans-radial amputees but neurologically intact. Jiang et al. [49], have used synergy based NMF techniques in order to simultaneously control two of the three degrees of freedom at the wrist joint. Contrarily brain damages might hamper our approach because of the resulted abnormal muscular activation and the consequent disruption of synergy formation: it has been found that synergies are either preserved or fractioned in neurologically impaired patients, depending on the extension of the brain lesion and the elapsed time from the stroke onset [32]. Therefore, we believe that extracting synergy information would represent a viable strategy for decoding user intentions even in a simultaneous classification scenario. The main focus of the proposed work was twofold: to highlight the importance of choosing both the extraction features and the decoding algorithm: we demonstrated that *synergy features* and ELM in the context of myoelectric decoding provided good results and we envision that they might be used in the specific context of assistive technology based on discrete action classification and user intention detection.

## Conclusion

The present paper focuses on comparing the two approaches in feature extraction techniques for real-time motion control of upper limb movements. Outcomes showed that the classification performance is better and more robust while using *synergy feature* than *EMG feature* sets.

Apart from contrasting the performance of these two types of control strategies, the aim of this investigation is to highlight the advantage of using the information provided by muscle synergies, to get a more accurate decoding of movements which involve a coordinated muscle activity. This approach would be highly beneficial for all those applications which rely on bio-signals in general(and not only EMG) to drive systems ranging from human augmentation to assistive technology, and this is particularly true when multiple degrees of freedom are involved. The use of ELM in synthesizing the decoder has provided tremendous advantages with the high rate of learning, and convergence to minimal error. In future we would like to incorporate the classification strategy in a soft wearable exosuit which is being designed for the upper limb [52, 53]. One drawback with this decoding scheme, is that the classification of motions is sequential and does not allow to classify a coordinated sequence of movements involving different joints. In future, it is our tenet to implement a simultaneous/parallel classification and using the classifier for assistive technology. It is worth mentioning that a robust algorithm for motion classification, would add an enormous benefit especially in those fields when a reduced muscular activity consequential to a neurological damage makes it difficult to detect the motor intention from the patient, and almost impossible to drive any kind of robotic device to support and provide assistance. That’s why we believe that using the proposed approach would bring additional trust and efficacy to those technologies which are mainly based on passive motion algorithm instead of boosting the capacity of detecting and discriminating motor intention.

## Abbreviations

ANN, artificial neural networks; EMG, electromyogram; ELM, extreme learning machine; LDA, linear discriminant analysis; MAV, mean absolute value; NMF, non-negative matrix factorization; SLFNN, single layer feed-forward neural network; SVM, support vector machines; VAR, variance

## Additional file

Additional file 1 Training and Testing phase. This video depicts the training phase, where a graphical user interface is used to instruct the subjects to perform specific motions for a specific period of time. The next section demonstrates the testing phase using *synergy features*, where EMG signals of the subject are used to drive the virtual arm to perform discrete motion tasks. https://www.youtube.com/watch?v=1riHz2TtT4o. (MP4 20582 kb)
